# Fine-Grained Image Classification for Crop Disease Based on Attention Mechanism

**DOI:** 10.3389/fpls.2020.600854

**Published:** 2020-12-22

**Authors:** Guofeng Yang, Yong He, Yong Yang, Beibei Xu

**Affiliations:** ^1^College of Biosystems Engineering and Food Science, Zhejiang University, Hangzhou, China; ^2^Agricultural Information Institute, Chinese Academy of Agricultural Sciences, Beijing, China; ^3^Key Laboratory of Agricultural Big Data, Ministry of Agriculture and Rural Affairs, Beijing, China

**Keywords:** crop disease, fine-grained, image classification, attention mechanism, fine-tuning

## Abstract

Fine-grained image classification is a challenging task because of the difficulty in identifying discriminant features, it is not easy to find the subtle features that fully represent the object. In the fine-grained classification of crop disease, visual disturbances such as light, fog, overlap, and jitter are frequently encountered. To explore the influence of the features of crop leaf images on the classification results, a classification model should focus on the more discriminative regions of the image while improving the classification accuracy of the model in complex scenes. This paper proposes a novel attention mechanism that effectively utilizes the informative regions of an image, and describes the use of transfer learning to quickly construct several fine-grained image classification models of crop disease based on this attention mechanism. This study uses 58,200 crop leaf images as a dataset, including 14 different crops and 37 different categories of healthy/diseased crops. Among them, different diseases of the same crop have strong similarities. The NASNetLarge fine-grained classification model based on the proposed attention mechanism achieves the best classification effect, with an *F*_1_ score of up to 93.05%. The results show that the proposed attention mechanism effectively improves the fine-grained classification of crop disease images.

## Introduction

Outbreaks of crop disease have a significant impact on the yield of agricultural production. Often, large-scale disease outbreaks destroy crops that have taken considerable efforts to grow, causing irreparable damage. Even without large-scale disease outbreaks, small-scale emergence can cause serious losses to crop yield and quality (Mutka and Bart, 2015). Therefore, developing techniques to accurately classify crop leaf disease categories is critical for disease prevention. With advances in image classification technology, researchers in the field of crop disease have gradually come to use deep learning approaches (Ramcharan et al., 2017; Fuentes et al., 2018; Liu B. et al., 2020). To date, research on the general classification of crop diseases has made several remarkable achievements in terms of better classification. However, for some fine-grained crop leaf diseases, there are still many difficulties.

Fine-grained image classification aims to classify sub-categories of a single larger category through fine-grained images (Peng et al., 2018). Examples include Stanford Cars (Yu et al., 2018; Tan and Le, 2019), CUB-200-2011 (Chen et al., 2019; Zhuang et al., 2020), FGVC Aircrafts (Ding et al., 2019; Sun et al., 2020), and Oxford 102 Flowers (Dubey et al., 2018; Touvron et al., 2019). Fine-grained image classification models can be divided into algorithms based on strong supervision and algorithms based on weak supervision, which depends on how much supervision information can be used. For classification models based on strong supervision information, superior classification accuracy during model training requires artificial annotation information, such as object bounding boxes and part annotation, in addition to image-level category labels. Fine-grained image classification models based on weakly supervised information are similar, but also require the use of global and local information. Weakly supervised fine-grained classification attempts to achieve better local information capture without resorting to the key point information of object parts. As our goal is fine-grained image classification, we need to build a model that can identify the most discriminating image features. Therefore, it is vital to detect subtle discriminatory features from similar regions (Ou et al., 2016; Zhang et al., 2016). Because the occurrence of crop diseases is often not controlled by humans, the fine-grained classification of crop diseases is common, but remains challenging. In general, different sub-categories have very similar appearance, although occasionally the different sub-categories are completely inconsistent. More seriously, the many visual disturbances (such as reflection, dispersion, and blur) caused by dew, shooting jitter, and light intensity seriously reduce the classification accuracy of crop disease images (Lu et al., 2017).

In terms of both theoretical research and practical applications, the fine-grained image classification of crop leaf diseases is of great importance, and is thus the focus of this study. Many researchers have studied the classification of crop diseases based on pattern recognition and machine learning. Guo et al. (2014) utilized texture and color features using a Bayesian approach for recognizing downy mildew, anthracnose, powdery, and gray mold infection with respective accuracy levels of 94.0, 86.7, 88.8, and 84.4%. Zhang et al. (2017) developed a leaf disease identification application in cucumber plants. This application isolates the infected part of the leaf through k-means clustering before extracting the color and shape, resulting in an accuracy level of 85.7%.

Although the above methods have made some progress, the identification and classification of diseases of different crops under actual field conditions can be further improved. For example, although some models can achieve extremely high accuracy on datasets under laboratory conditions, they often have poor identification effects when faced with actual field conditions. We think this is because insufficient disease features are extracted, resulting in a lack of disease details. In summary, the main challenge of fine-grained image classification of crop leaf diseases is undoubtedly the subtle discrimination between different sub-categories. The primary difficulties can be roughly divided into three aspects: (1) the similarity between the sub-categories under the same disease category is very strong; (2) the field environment has significant background interference; and (3) the location of different crop diseases is inconsistent.

In an attempt to overcome these difficulties, many researchers have applied convolutional neural network (CNN) to crop disease classification. To investigate the impact of dataset size and species on the effectiveness of crop disease classification based on deep learning and transfer learning, Barbedo (2018) showed that, although CNNs can largely overcome the technical limitations associated with automated crop disease classification, training with a limited set of image data can have many negative consequences. Kaya et al. (2019) studied and demonstrated that the transfer learning model can help crop classification identification and improve the low-performance classification model. Too et al. (2019) fine-tuned and evaluated the most advanced deep CNN for image-based crop disease classification. The data used in their experiments covered 38 different categories, including disease and health images of the leaves of 14 crops from PlantVillage. The accuracy of DenseNet reached 99.75%, better than that of other models. Cruz et al. (2019) used CNNs to detect leaf images of Grapevine Yellows (GY) disease in red vines (cv. Sangiovese). ResNet-50 was found to be the best compromise network in terms of accuracy and training cost. Turkoglu et al. (2019) proposed a multi-model pre-trained CNN (MLP-CNN) based on long short-term memory for detecting apple diseases and insect pests. Their results were comparable to or better than those of pre-trained CNN models. Deep learning has been widely applied to various crop categories and crop disease classification studies, and deep learning models based on transfer learning can accelerate the training stage. At the same time, to cope with the impact of complex scenes on model classification performance, it is necessary to enhance the performance of CNNs to better handle fine-grained image classification tasks.

In recent years, it has been found that human cognitive processes do not focus attention on the entire scene at one time. On the contrary, they pay more attention to local regions in the scene while extracting relevant information. Models based on attention mechanisms have achieved good results on many challenging tasks, such as visual question answering (Malinowski et al., 2018), object detection (Li et al., 2019), and scene segmentation (Fu et al., 2019). Although the attention mechanism has been applied to different tasks, it has not been used for the fine-grained classification of crop disease images.

In this research, we propose a novel attention mechanism and use transfer learning to quickly build several fine-grained image classification models of crop diseases based on the attention mechanism, so as to solve the problem that the accuracy of CNN model in complex scenes is low due to visual interference in practical applications. Therefore, the contributions of this paper are as follows:

According to the characteristics of crop disease images in real scenes, a fine-grained fine-tuning classification algorithm based on attention mechanism is constructed on the basis of using pre-trained CNN to extract convolutional features of fine-grained images as the input of the network. The attention mechanism makes the classification algorithm pay more attention to some more discriminative local regions of the image, thereby improving the classification accuracy of the model in complex scenes.

We collect crop disease images in real scenes and added these images to the PlantVillage dataset to form a new hybrid dataset for training the CNN model. We verify the effectiveness of our proposed method by designing multiple comparative experiments.

In addition, we also explore and prove the importance of the image source to the classification results, as well as the impact of problem scenes and special interference on the classification results.

The rest of this paper is organized as follows. Section Materials and methods introduces the main experimental dataset and our proposed fine-grained fine-tuning classification algorithm based on the attention mechanism. The experimental results are described in section Results. In section Discussion, we discuss the importance of training image sources, the impact of problem scenes and special interference on the classification results. Finally, this paper concludes in Section conclusion.

## Materials and Methods

### Image Datasets

**Figure 1:**
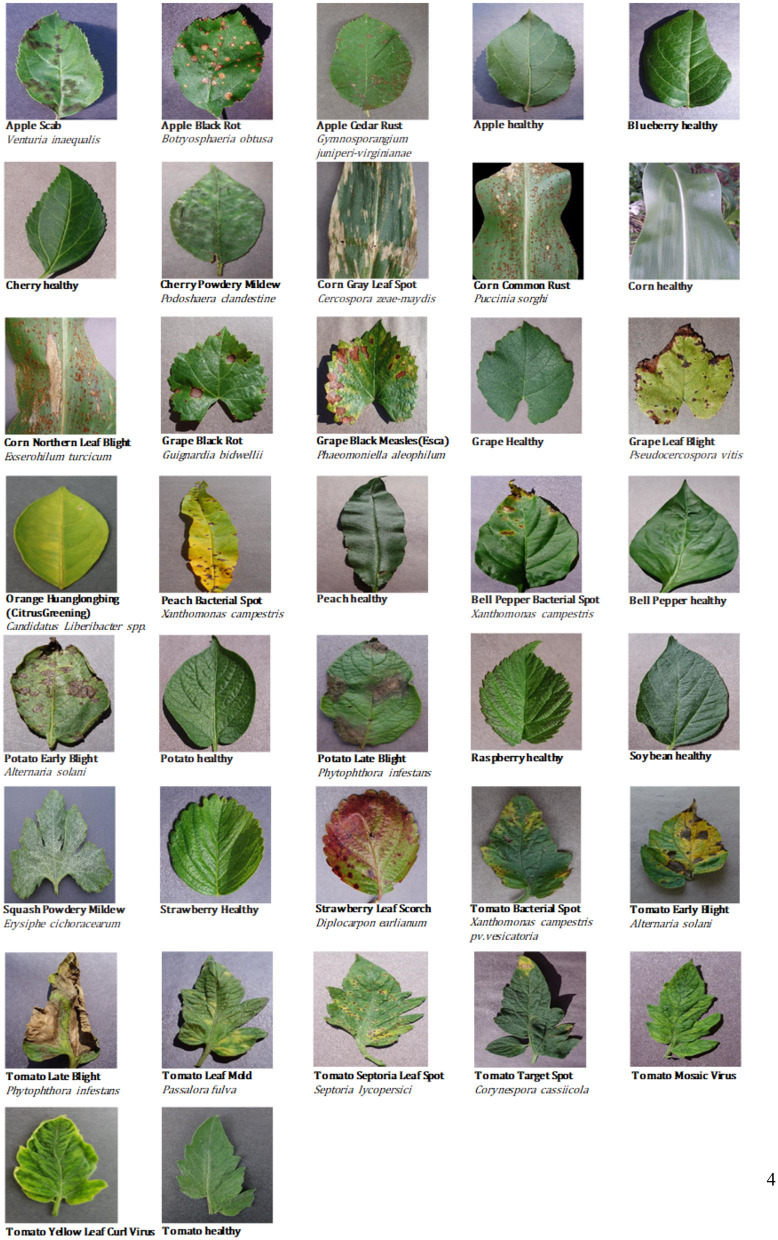
Examples of crop leaf images in the dataset.

This study considered the PlantVillage public dataset of 52,629 images (except for images from the Tomato Two Spotted Spider Mite, Tetranychus urticae, category) (Mohanty et al., 2016), which covers a total of 37 categories including images of 14 different healthy or diseased crops. Using the Scrapy web crawler on the Internet's agricultural technology and consulting platforms, we then extracted a total of 5,571 images uploaded by users in the abovementioned 37 categories (images of crop diseases under actual field conditions). Finally, CNN models were trained and tested using the full dataset of 58,200 healthy and diseased crop disease images (Figure 1).

Table 1 provides statistical data for the 37 categories of the dataset, such as the number of images for each category and the percentage of images taken under laboratory or field conditions. It is well-known that there is a single color or no background in the image of laboratory conditions, while the background in the image of field conditions is relatively complex and changeable. As shown in Table 1, nearly 10% of the available images were taken under field conditions.

**Table 1:** Statistics of crop healthy/diseased images and related data.

**Class**	**Plant common name**	**Disease common name**	**Disease scientific name**	**Images (PlantVillage)**	**Laboratory conditions (%)**	**Field conditions (%)**
1	Apple	Apple scab	*Venturia inaequalis*	800 (630)	78.75%	21.25%
2	Apple	Black rot	*Botryosphaeria obtuse*	800 (621)	77.63%	22.38%
3	Apple	Cedar apple rust	*Gymnosporangium juniperi-virginianae*	400 (275)	68.75%	31.25%
4	Apple	—	—	1,800 (1,645)	91.39%	8.61%
5	Blueberry	—	—	1,700 (1,502)	88.35%	11.65%
6	Cherry (and sour)	—	—	1,000 (854)	85.40%	14.60%
7	Cherry (and sour)	Powdery mildew	*Podosphaera* spp.	1,200 (1,052)	87.67%	12.33%
8	Com (maize)	Cercospora leaf spot	*Cercospora zeae-maydis*	700 (513)	73.29%	26.71%
9	Com (maize)	Common rust	*Puccinia sorghi*	1,300 (1,192)	91.69%	8.31%
10	Com (maize)	—	—	1,300 (1,162)	89.38%	10.62%
11	Com (maize)	Northern Leaf Blight	*Exserohilum turcicum*	1,100 (985)	89.55%	10.45%
12	Grape	Black rot	*Guignardia bidwellii*	1,300 (1,180)	90.77%	9.23%
13	Grape	Esca (Black measles)	*Phaeomoniella chlamydospora*	1,500 (1,383)	92.20%	7.80%
14	Grape	—	—	600 (423)	70.50%	29.50%
15	Grape	Leaf blight	*Pseudocercospora vitis*	1,200 (1,076)	89.67%	10.33%
16	Orange	Huanglongbing	Candidatus Liberibacter	5,700 (5,507)	96.61%	3.39%
17	Peach	Bacterial sport	*Xanthomonas campestris*	2,400 (2,297)	95.71%	4.29%
18	Peach	—	—	500 (360)	72.00%	28.00%
19	Pepper, bell	Bacterial spot	*Xanthomonas campestris*	1,100 (997)	90.64%	9.36%
20	Pepper, bell	—	—	1,600 (1,478)	92.38%	7.63%
21	Potato	Early blight	*Altemaria solani*	1,200 (1,000)	83.33%	16.67%
22	Potato	—	—	300 (152)	50.67%	49.33%
23	Potato	Late blight	*Phytophthora infestans*	1,200 (1,000)	83.33%	16.67%
24	Raspberry	—	—	500 (371)	74.20%	25.80%
25	Soybean	—	—	5,200 (5,090)	97.88%	2.12%
26	Squash	Powdery mildew	*Erysiphe cichoracearum, Sphaerotheca fuliginea*	2,000 (1,835)	91.75%	8.25%
27	Strawberry	—	—	600 (456)	76.00%	24.00%
28	Strawberry	Leaf scorch	*Diplocarpon earlianum*	1,300 (1,109)	85.31%	14.69%
29	Tomato	Bacterial spot	*Xanthomonas campestris* pv. Vesicatoria	2,300 (2,127)	92.48%	7.52%
30	Tomato	Early blight	*Altemaria solani*	1,200 (1,000)	83.33%	16.67%
31	Tomato	Late blight	*Phytophthora infestans*	2,100 (1,909)	90.90%	9.10%
32	Tomato	Leaf Mold	*Fulvia fulva*	1,100 (952)	86.55%	13.45%
33	Tomato	Septoria leaf spot	*Septoria lycopersici*	1,900 (1,771)	93.21%	6.79%
34	Tomato	Target spot	*Corynespora cassiicola*	1,600 (1,404)	87.75%	12.25%
35	Tomato	Tomato mosaic virus	*Tomato mosaic virus* (ToMV)	500 (373)	74.60%	25.40%
36	Tomato	TYLCV	Begomovirus (Fam. Geminiviridae)	5,500 (5,357)	97.40%	2.60%
37	Tomato	—	—	1,700 (1,591)	93.59%	6.41%
TOTAL:				58,200 (52,629)	90.43%	9.57%

*Images column gives the number of images in each category, where the figures in parentheses are from the public dataset*.

**Figure 2:**
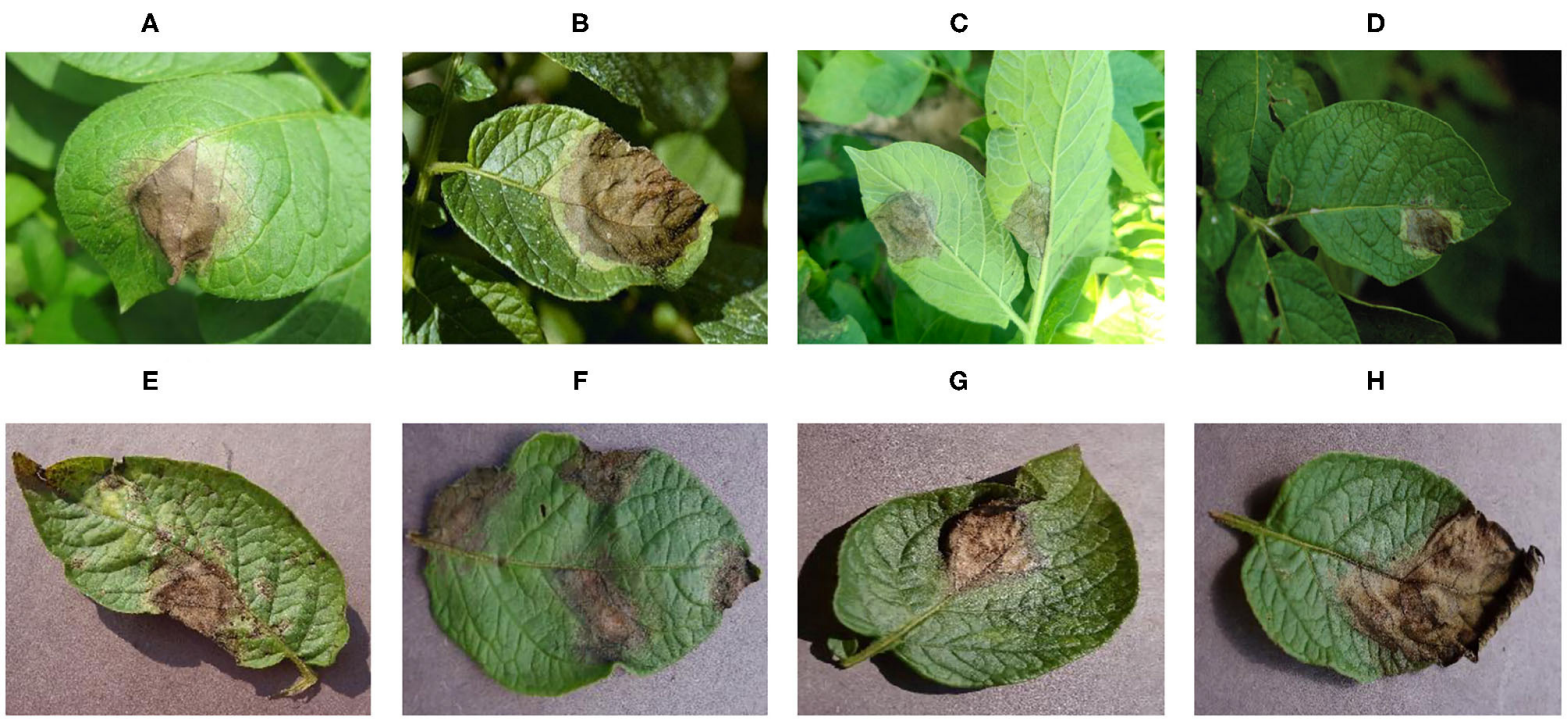
Image sample of potato late blight. **(A–D)** Field conditions, **(E–H)** laboratory conditions.

Figure 2 shows disease images of potato late blight, including four disease images obtained under field conditions and four disease images obtained under laboratory conditions. The increase in complexity of the four diseased images under field conditions is obvious (e.g., there are many leaves and other parts in the images, different backgrounds, shadow effects, and so on).

The dataset includes images taken under laboratory conditions and under field conditions (see Figure 2); the percentages of each are presented in Table 1. The whole dataset was randomly divided into a training set (80%) and a test set (20%). Therefore, 46,560 images were used for CNN model training, while the remaining 11,640 images were used to test the performance of the model. The training set and the test set were preprocessed to satisfy the model's input size requirements, and the image sizes were reduced and cropped to 256×256, 299×299, and 331×331 pixels.

We conducted several experiments to evaluate the importance of the conditions under which the leaf images were captured. Namely, we first conducted the training using only laboratory conditions images (PlantVillage, 52,629 photos) and the testing using images of actual field conditions (Internet, 5,571 photos), and then performed training using only images of actual field conditions (Internet, 5,571 photos) and testing with images taken under laboratory conditions (PlantVillage, 52,629 photos).

### Experimental Methods and Parameters

#### Transfer Learning

VGG, ResNet, and other deep CNN models have achieved great success in image classification. The pre-trained deep CNN model has been fully trained on a large image dataset (ImageNet), allowing many features required for image classification to be learned. Therefore, we can use the idea of transfer learning to fully utilize the large amount of knowledge learned by pre-training the CNN model on the ImageNet dataset, and apply it to crop disease image classification. This paper describes how the transfer learning method of parameter transfer was adopted to remove the maximum pooling and fully connected layers after the final convolution, and introduces a new fine-grained classification model based on the attention mechanism. Compared with the random initialization of the weight parameters of each layer of the network, the fine-tuning method helps accelerate the convergence of the network.

For image classification, there are several CNN baseline models that have been successfully applied to specific tasks. Regarding the task of image recognition and classification of crop diseases, six CNN models that were pre-trained using ImageNet have been applied: (1) VGG16 and VGG19 (Simonyan and Zisserman, 2015), (2) ResNet50 (He et al., 2016), (3) InceptionV3 (Szegedy et al., 2016), (4) Xception (Chollet, 2017), and (5) NASNetLarge (Zoph et al., 2018). The training and testing processes of these pre-trained models and of the proposed fine-grained pre-trained model based on the attention mechanism were implemented using the TensorFlow machine learning computing framework. Model training and testing was conducted with four NVIDIA Tesla V100 GPUs.

#### Attention Mechanism

The attention mechanism was first applied to natural language processing. It is often combined with recurrent neural networks, resulting in good prediction and processing ability for text sequences. In recent years, the attention mechanism has also been widely used for image classification (Meng and Zhang, 2019; Xiang et al., 2020), object detection (Chen and Li, 2019; Xiao et al., 2020), and image description generation (Liu M. et al., 2020; Zhang et al., 2020). In the field of crop disease classification, most researchers have tended to use transfer learning technology. There has also been some research on crop disease identification based on the attention mechanism (Nie et al., 2019; Karthik et al., 2020). These previous studies have focused on a certain crop, and so the disease category and scale of the dataset are limited. Therefore, we conducted related experiments to verify that increasing the attention mechanism can improve the effect of crop disease classification based on transfer learning technology.

The attention of an image refers to the process of obtaining a target region that requires attention as the human eye rapidly scans the global image. This target region is assigned more attention (weight distribution) to obtain the required detailed information about the target, with other useless information suppressed. Soft attention is most commonly used, because this is a completely differentiable process that can realize end-to-end learning in CNN models. Most soft attention models learn an attention template to align the weights of different regions in a sequence or an image and use this template to locate the distinguishable regions. Different from soft attention, the hard attention mechanism is a random, non-differentiable process that determines the importance of individual regions one at a time, rather than identifying the important regions within the whole image.

**Figure 3:**
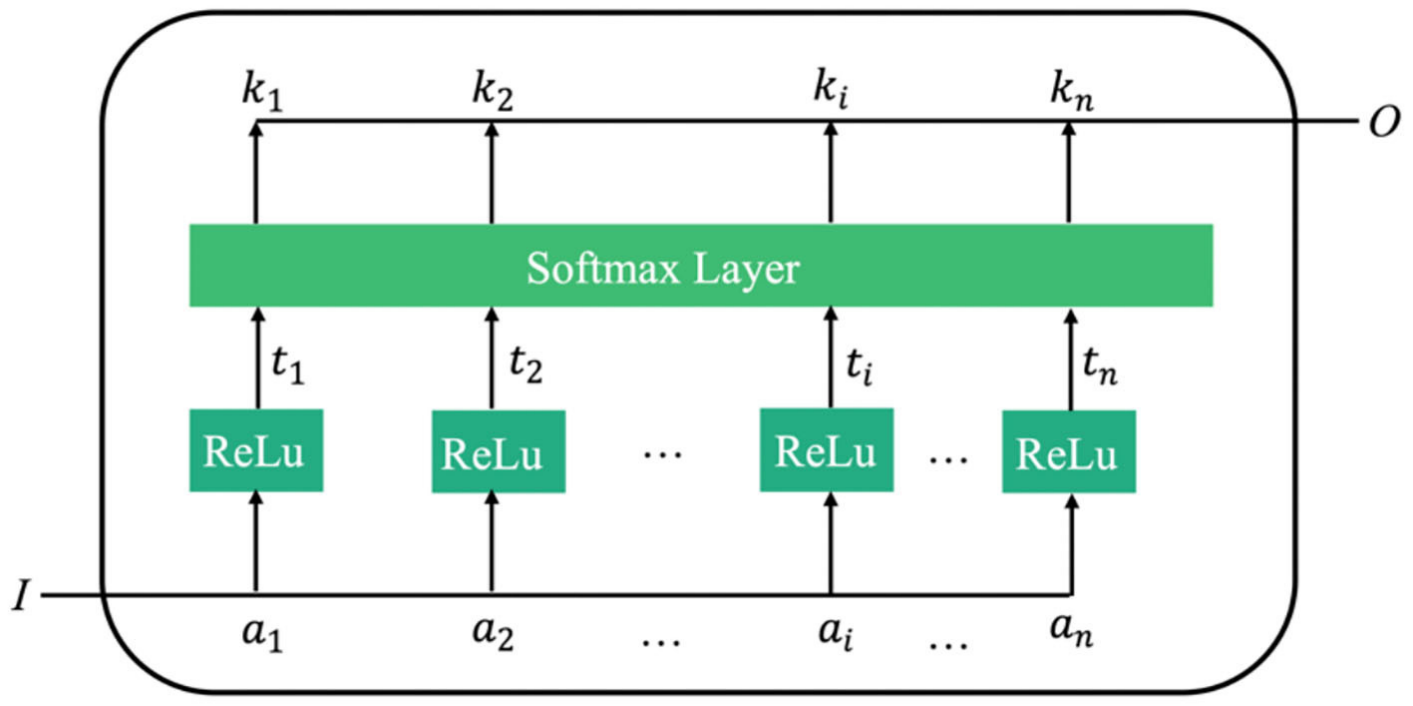
Schematic diagram of learning mechanism of attention.

For image classification, the weight of the arithmetic mean of attention can be extracted through attention learning to form the attention spectrum of the image. Similar to traditional natural language processing, the image-based attention can be obtained through the model illustrated in Figure 3.

In Figure 3, *I* is the input image. The attention model has *n* parameters, *a*_1_, *a*_2_, ⋯ , *a*_*i*_, *a*_*n*_, which respectively represent a description of each part of the image. *O* is the return value of the model's attention spectrum (more specifically, the weight values of the *n* parameters), which is determined from the importance of each *a*_*i*_ relative to the input *I*. By filtering the input image through this output, the region that requires most attention can be identified.

#### Proposed Model

**Figure 4:**
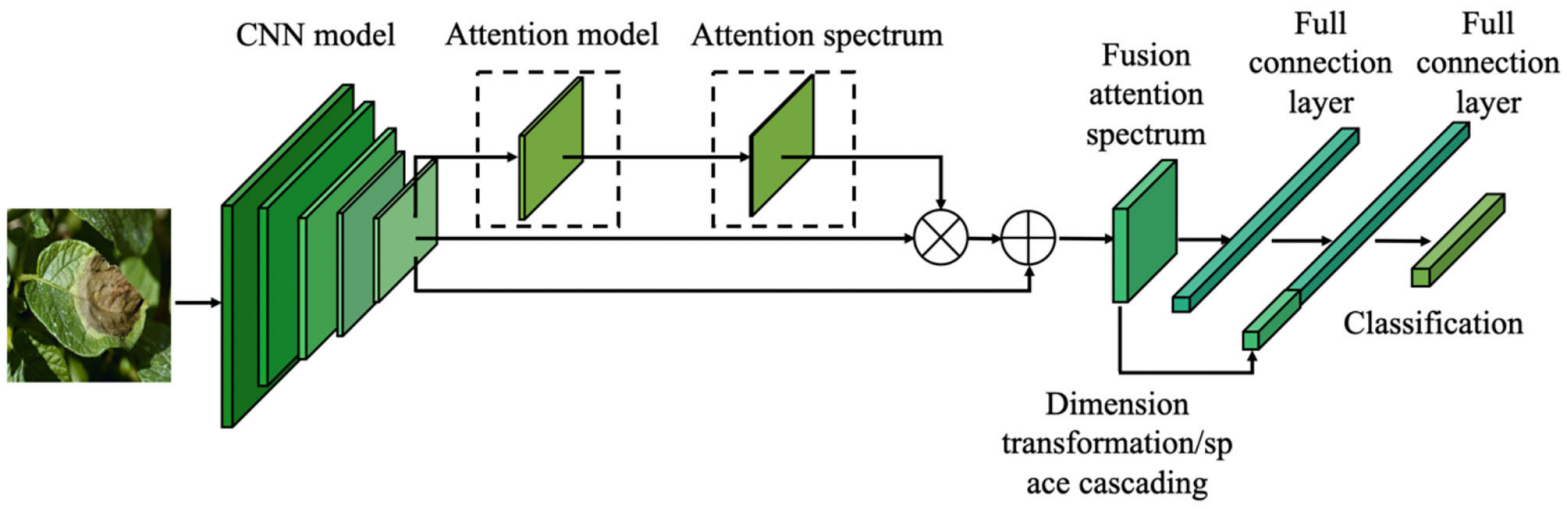
Fine-grained image classification network structure of crop diseases based on attention mechanism.

Based on the pre-trained model described in Section transfer learning and the attention mechanism introduced in Section attention mechanism, this paper proposes a fine-grained classification model based on the attention mechanism (Figure 4). By learning the attention of the CNN feature spectrum, the attention model calculates and identifies the most important region of the feature spectrum for the final classification task, and provides the maximum attention input (weight distribution). However, adding the attention weight to the last layer of the CNN features will cause different degrees of suppression of the original features. To overcome this suppression, the weighted feature spectrum is added to the original feature spectrum. The fusion spectrum is then input into the fully connected layer. In the second fully connected layer, the attention feature spectrum transformed by the global average pooling dimension is connected with the fully connected feature spectrum in the channel direction, before being sent to the classification layer for classification.

The attention model proposed in this paper adopts an unsupervised training mode. There is no pre-labeled ground truth to constrain the attention spectrum, so there is no separate loss calculation. Instead, a backpropagation adaptive mode is used to constrain the weight distribution of attention. The loss function defined in this paper is expressed as:

(1) $$\begin{array}{l} loss=\frac{1}{2n}\sum {||y_{truth}\left(x\right)-y_{pred}\left(x\right)||}^{2} \end{array}$$

where *n* = 37 is the number of input samples, *x* is the input sample, *y*_*truth*_ is the actual category, and *y*_*pred*_ is the predicted category output by the final layer of the network. In the process of backpropagation, the output error of the *Softmax* layer is backpropagated, and the parameters are updated using the random gradient descent method, so that the final loss function value decreases and the network converges.

Through the soft attention mechanism, the output of the final convolutional layer of the CNN is obtained. This is taken as the input of the attention model, and the corresponding attention spectrum is calculated. The original feature spectrum is then weighted by the attention spectrum, and the output attention feature spectrum is provided as the input for the subsequent network. Let the feature spectrum of the output of the final convolution spectrum after the pooling operation be expressed as *f* ∈ ℝ^*H*×*W*×*C*^, where *H* and *W* refer to the height and width of the feature spectrum of this layer, *C* refers to the number of channels of the feature spectrum of this layer, and for each position (*m, n*) on the spectrum, its feature value is expressed as $f_{m,n}\epsilon {\mathbb{R}}^{C}$. The corresponding attention weight *W*_*m,n*_ can then be obtained as:

(2) $$\begin{array}{l} W_{m,n}=ATT\left(f_{m,n};W_{att}\right) \end{array}$$

where *ATT* is a mapping function learned by the attention model and *W*_*att*_ is the weight parameter of the attention model. Through *Softmax* regression of *w*_*m,n*_, the final attention spectrum *M* = [*M*_*m,n*_] is obtained as a normalized probability matrix, where *M*_*m,n*_ is expressed as:

(3) $$\begin{array}{l} M_{m,n}=Softmax\left(W_{m,n}\right) \end{array}$$

**Figure 5:**
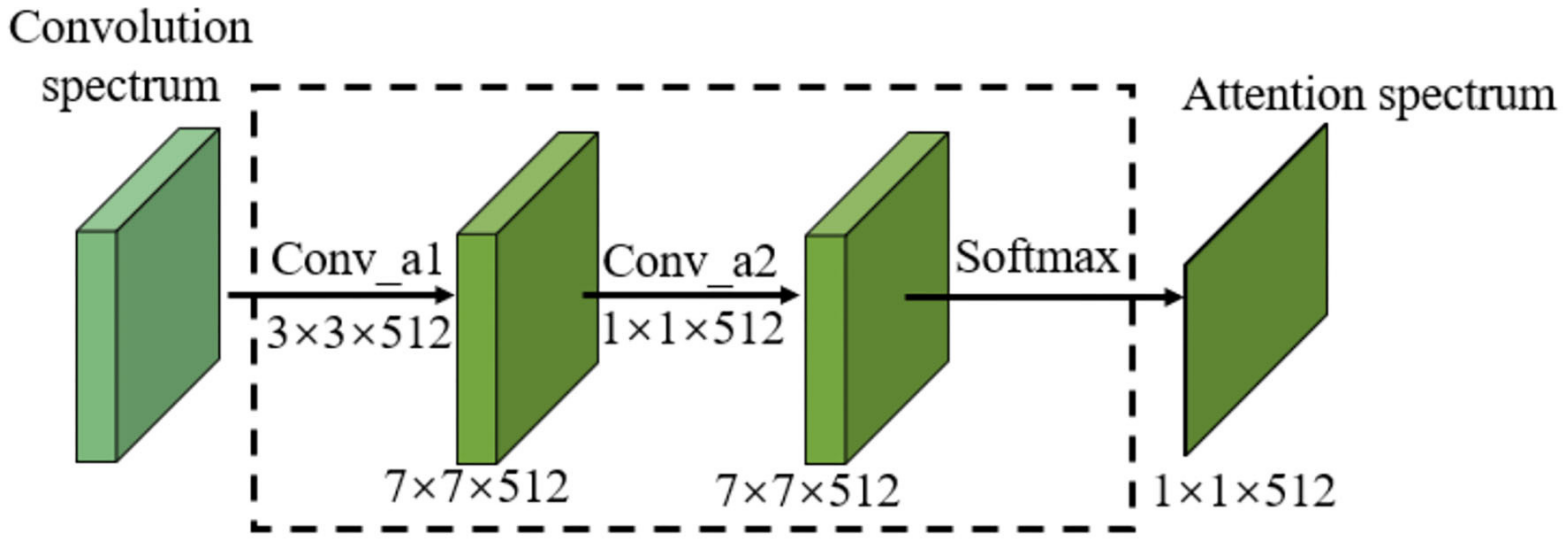
Attention calculation model for images.

As can be seen from Figure 5, the attention model proposed in this paper takes the output of the final convolution spectrum in the neural network as its input. The attention model includes two convolutional layers and one *Softmax* layer. The kernel sizes of the convolutional layers are 3×3 and 1×1. The attention feature spectrum $f^{att}=\;\left[f_{m,\;n}^{att}\right]$ is obtained by multiplying the attention spectrum *M* by the CNN feature spectrum *f*, and is expressed as

(4) $$\begin{array}{l} f^{att}=\left[f_{m,n}^{att}\right]=\left[M_{m,n}\cdot f_{m,n}\right] \end{array}$$

The 3×3 convolution kernel further extracts the CNN feature of the final convolution spectrum. In order not to reduce the feature receptive field and feature information, a convolution kernel with the same size as the original network is selected. Compared with the 3×3 convolution kernel, the 1×1 convolution kernel enables information interaction and integration across channels. By connecting features in the channel direction, nonlinear components can be added to features to improve the feature expression ability of the attention model.

The attention spectrum of the final convolution spectrum of the CNN is obtained through the attention model, and the attention spectrum and the original CNN feature spectrum are then multiplied to obtain the attention feature spectrum. The attention spectrum is the spectrum obtained after normalizing the weights of the features. According to this definition, the attention feature spectrum obtained after multiplication has a certain attenuation compared with the original CNN feature spectrum. Additionally, during the convolution and probability calculation, the spatial transformation of the CNN feature spectrum and noise addition means that the calculated attention spectrum may be distorted. In this case, the obtained attention spectrum has no guiding significance for the original image spectrum. To overcome this problem, once the attention feature spectrum has been obtained, the original CNN feature spectrum is added and fused to obtain the final attention $f_{all}^{att}$, which is input into the subsequent fully connected layer, as shown in Equation (5).

(5) $$\begin{array}{l} f_{all}^{att}\;=\;f+M\cdot f \end{array}$$

By adding the attention spectrum to the CNN feature spectrum, the distortion of the attention spectrum is overcome and the original feature spectrum before the fully connected layer can be effectively utilized.

**Figure 6:**
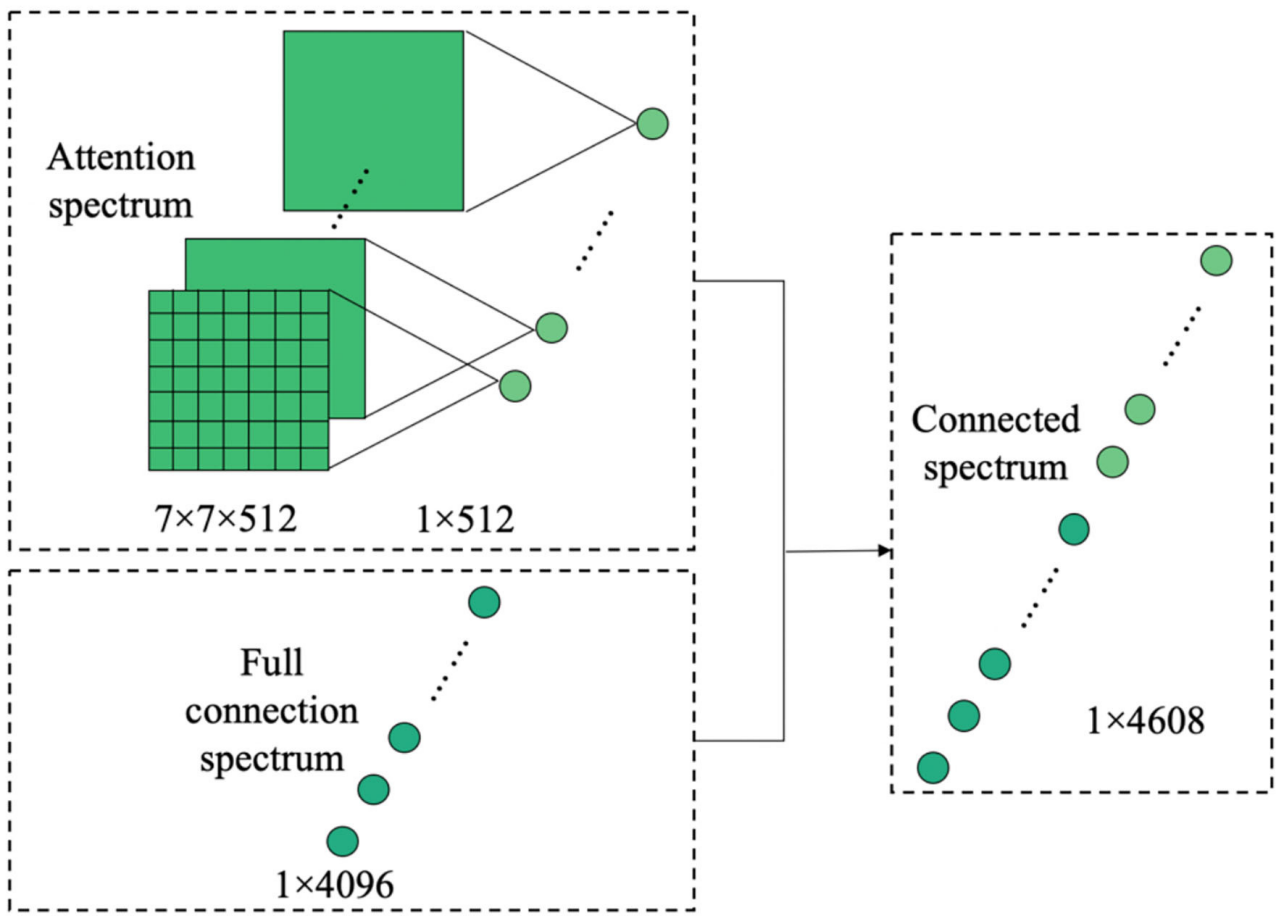
Dimension transformation and connection diagram of attention feature spectrum.

By extracting and merging the attention spectrum, a spectrum of features is obtained that is well-located and noticeable in space. This spectrum is then input to the subsequent fully connected layer. As the connection operation maps the convolutional spectra of all channels to one point in the fully connected layer, the spatial information is destroyed by the operation of the fully connected layer. The original intention of introducing the attention model is to extract and improve the significant regions of the CNN feature spectrum in space. However, after the fully connected layer, the spatial information of the extracted attention feature has also been destroyed. Therefore, the attention space feature is reused by connecting the attention feature spectrum in the final fully connected layer, as shown in Figure 6.

### Evaluation of the Model

The accuracy, precision (P), recall (R), and comprehensive *F*_1_ evaluation index were used to evaluate the crop disease image classification model. The *F*_1_ value is the harmonic average of the precision and recall, and has a maximum of 1 and a minimum of 0. It is calculated as follows:

(6) $$\begin{array}{l} F_{1}=\frac{2PR}{P+R}\times 100\% \end{array}$$

### Experimental Details

In all our experiments, we preprocessed the images to sizes of 256×256, 299×299, and 331×331 pixels, conducted a total of 1,000 training epochs, and used a batch size of 32. We used a momentum SGD initial learning rate of 0.001. When the standard evaluation stopped increasing, the learning rate was multiplied by 0.1 until it had dropped to 0.0001. After lowering the learning rate, we waited for five epochs before returning to normal operation. If the loss of the test set did not improve after 20 epochs, the learning rate was reduced. We conducted experiments using multiple pre-trained models, all of which are robust to the selection of hyper-parameters.

## Results

### Compared With the Pre-training Model and the Effect of the Attention Mechanism

Tables 2, 3 present the classification accuracy, precision, recall, and *F*_1_ values of various models on the test set. The results indicate that the fine-grained fine-tuning classification models based on the attention mechanism outperform the original pre-trained models by 1–2% in terms of accuracy, precision, recall, and *F*_1_ value. This demonstrates that the attention mechanism improves the classification performance of the models and allows them to focus on key regions in the image. The fine-grained NASNetLarge model based on the fine-grained attention mechanism achieves the highest accuracy, precision, recall, and *F*_1_ values, and thus provides the best classification performance. These 12 models were further trained using only the original image to record the training period for the best performance. As shown in Table 3, the fine-grained NASNetLarge model based on the attention mechanism achieves the highest classification accuracy of 95.62%. Thus, this model was used in subsequent experiments for crop disease image classification.

**Table 2:** Results of pre-trained model on test set.

**Pre-trained model**	**Size(MB)**	**Accuracy (%)**	**Precision (%)**	**Recall (%)**	**F1-measure (%)**	**Parameters**
VGG16 (Simonyan and Zisserman, 2015)	56.16	83.07	82.52	80.13	81.31	15,360,589
VGG19 (Simonyan and Zisserman, 2015)	76.42	85.79	83.45	81.75	82.59	20,670,285
ResNet50 (He et al., 2016)	90.38	85.82	85.21	83.63	84.41	25,769,613
InceptionV3 (Szegedy et al., 2016)	83.84	88.47	87.78	85.47	86.61	23,984,685
Xception (Chollet, 2017)	79.81	91.22	90.24	87.05	88.62	23,043,381
NASNetLarge (Zoph et al., 2018)	327.69	92.78	92.16	90.83	91.49	89,082,719

**Table 3:** Results of fine-grained classification model based on attention on test set.

**Model based on attention mechanism**	**Size(MB)**	**Accuracy (%)**	**Precision (%)**	**Recall (%)**	**F1-measure (%)**	**Parameters**
VGG16*	59.23	85.53	84.68	81.32	82.97	15,514,783
VGG19*	79.75	86.40	84.93	82.05	83.47	20,823,653
ResNet50*	93.91	87.03	86.27	85.32	85.79	25,942,746
InceptionV3*	87.53	90.64	88.51	88.48	88.49	24,173,257
Xception*	83.28	92.89	91.82	90.95	91.38	23,218,425
NASNetLarge*	331.75	95.62	94.35	91.79	93.05	89,286,814

The * indicates that the pre-training model is used.

**Figure 7:**
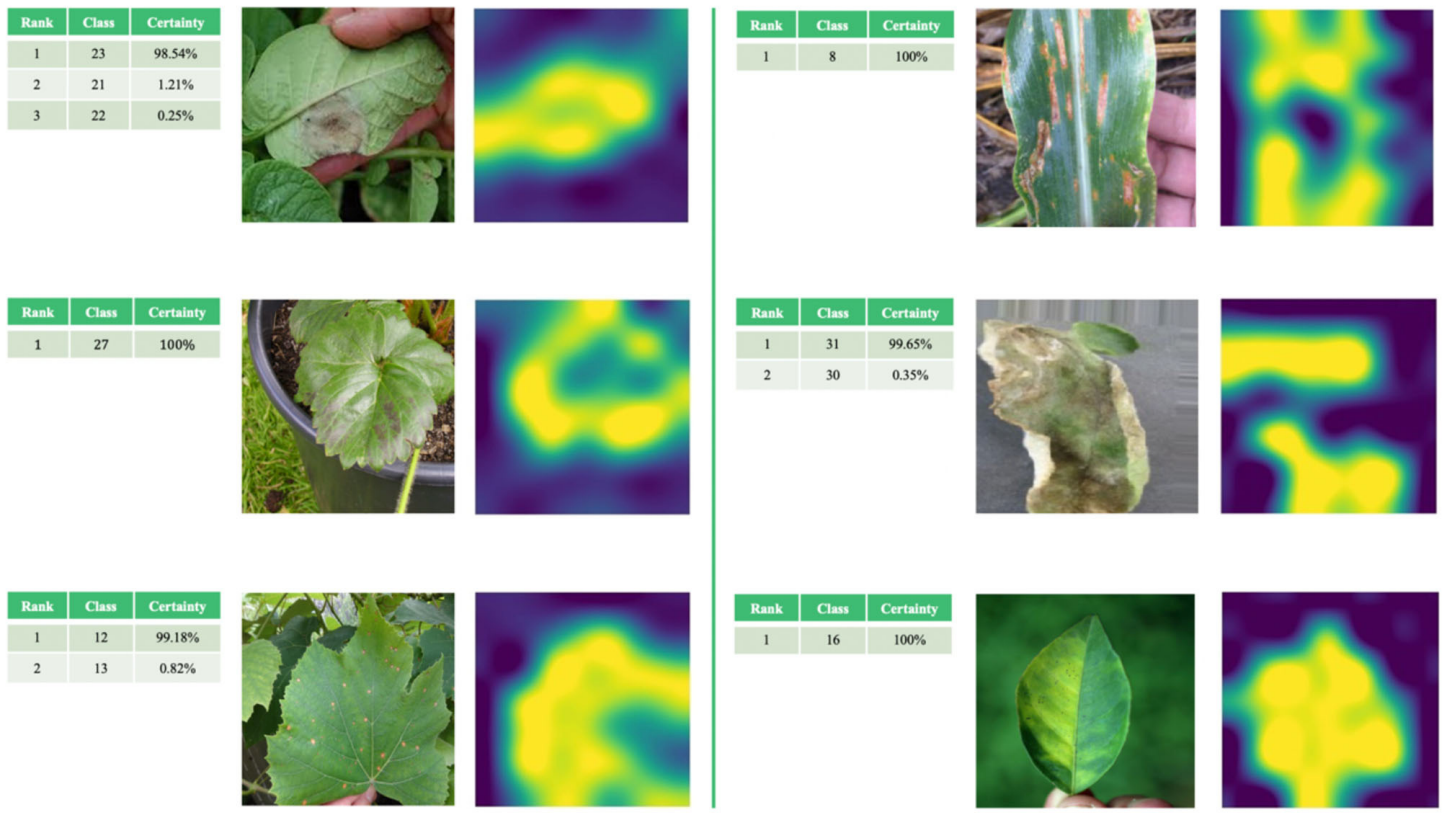
Examples of correct classification of test set images and visualization of attention mechanism.

Figure 7 provides a visual representation of some random images from the test set. The table on the left of the original image shows the predicted classification. The image to the right of the original image is a visual representation of the attention mechanism using the fine-grained fine-tuning NASNetLarge model based on the attention mechanism. The highest-ranked classification result for each image was considered as the final classification result predicted by the model. The images of the crop leaves shown in Figure 7 are correctly classified. In most cases, the degree of certainty for the correct classification is close to 100%, so there is no actual ranking.

### Testing on Different Dataset

We also comprehensively evaluated our algorithm on public plant datasets of Flavia (Wu et al., 2007), Swedish Leaf (Söderkvist, 2001), and UCI Leaf (Silva et al., 2013). These datasets contain clear images, and they are widely used datasets in this field, often used for algorithm development and comparison. The statistics of three datasets are shown in Table 4. We follow the same training/test split as in Section image datasets.

**Table 4:** Statistics of benchmark datasets.

**Datasets**	**Class**	**Train**	**Test**
Flavia	32	1,526	381
Swedish leaf	15	900	225
UCI leaf	40	356	87

The Flavia dataset contains 1,907 images of 32 species of plants. All images in the dataset have a white background, and the number of each category varies from dozens of images and is relatively unbalanced.

The Swedish Leaf dataset contains 15 plant species, with 75 images in each category. All plant leaf images are images with white background, and the quality and resolution of each image is high.

The UCI Leaf dataset contains 40 different plants and a total of 443 images. The background colors of the images in this dataset are all pink. The number of images in each category ranges from a few to a dozen.

As seen from Table 5 the NASNetLarge model based on the attention mechanism constructed by our proposed method can still get the best classification accuracy on the three public plant datasets. Therefore, it can be proved that our model has better performance across datasets and can achieve efficient classification on datasets of different sizes.

**Table 5:** Comparison of methods for image classification in three datasets.

**References**	**Datasets**	**Accuracy/%**	**Method**
Lee et al. (2017)	Flavia	99.40	CNN, Fine-tuning
Yousefi et al. (2017)	Flavia	97.50	Fourier and Wavelet Descriptors, MLP
Murat et al. (2017)	Flavia	95.25	HOG, Moments, ANN,
	Swedish	99.89	RF, and SVM
Kaya et al. (2019)	Flavia	99.00	DF – VGG16/LDA
	Swedish	98.80	CNN – RNN
	UCI Leaf	96.20	DF – Alexnet/LDA
Our	Flavia	99.72	NASNetLarge –
	Swedish	99.90	Attention
	UCI Leaf	98.74	

### Validation and Comparison of Proposed CNN With Traditional Machine Learning Models

The traditional machine learning methods used for comparison in this paper are SVM, Decision tree, k-NN, and Naive Bayes. Features such as Hu-moments, Haralick features, LBP features, and HSV features have been used to evaluate the performance of all traditional Machine Learning algorithms. The results are given in Table 6.

**Table 6:** Comparison of methods for image classification in three datasets.

**Models**	**Features**	**Accuracy/%**	**Precision/%**	**Recall/%**	**F1-measure/%**
Naïve Bayes	Haralick, Hu	31.67	17.11	12.23	14.26
	Hu-moments, HSV	35.61	20.92	15.56	17.84
	Haralick, Hu, HSV	39.08	29.33	20.55	24.17
	Haralick, Hu, HSV, LBP	43.24	36.76	25.64	30.21
Decision tree	Haralick, Hu	41.33	23.24	18.85	20.82
	Hu-moments, HSV	47.18	30.52	25.21	27.61
	Haralick, Hu, HSV	53.91	43.45	34.69	38.58
	Haralick, Hu, HSV, LBP	60.23	48.32	41.36	44.57
SVM	Haralick, Hu	43.33	30.42	23.28	26.38
	Hu-moments, HSV	52.67	41.23	33.01	36.66
	Haralick, Hu, HSV	55.82	45.35	36.82	40.64
	Haralick, Hu, HSV, LBP	61.45	54.51	43.24	48.23
k-NN	Haralick, Hu	69.66	65.23	53.72	58.92
	Hu-moments, HSV	75.38	73.57	61.34	66.90
	Haralick, Hu, HSV	79.52	77.41	66.38	71.47
	Haralick, Hu, HSV, LBP	84.45	80.65	74.77	77.60
Our model (NASNetLarge*)		95.62	94.35	91.79	93.05

The * indicates that the pre-training model is used.

Table 6 shows that the accuracy, precision, recall and *F*_1_ of our proposed model are much higher than those obtained using other machine learning algorithms.

## Discussion

### Importance of Training Image Type

The fine-grained NASNetLarge model based on the attention mechanism produced the best classification effect, and was therefore further tested to study the importance of assessing the conditions under which the leaf image was captured. The corresponding results are presented in Table 7.

**Table 7:** Results of using different training sets and test sets with the optimal model under laboratory conditions and field conditions.

**Model based on attention mechanism**	**Training: laboratory** **Testing: actual field conditions**				**Training: actual field conditions** **Testing: laboratory**			
	**Accuracy (%)**	**Precision (%)**	**Recall (%)**	**F1-measure (%)**	**Accuracy (%)**	**Precision (%)**	**Recall (%)**	**F1-measure (%)**
NASNetLarge*	38.52	34.28	33.95	34.11	66.85	61.32	59.64	60.47

The * indicates that the pre-training model is used.

When only laboratory or field condition images are used for training, the accuracy on the test set is significantly lower than when both laboratory and field condition images are used for training. The results show that the model can obtain better performance when using images obtained under field conditions for training and requiring classification of laboratory condition images (*F*_1_ value is nearly 60.47%). In contrast, when the laboratory condition images are used for training and the field condition images are classified, the classification performance is obviously reduced (*F*_1_ value is about 34.11%). This shows that image classification under field conditions is a more difficult and complicated task than the classification of images obtained under laboratory conditions, and proves that the construction of an efficient automatic detection and diagnosis system for crop diseases using images obtained under field conditions is of great significance.

### Problematic Situations and Indicative Cases

**Figure 8:**
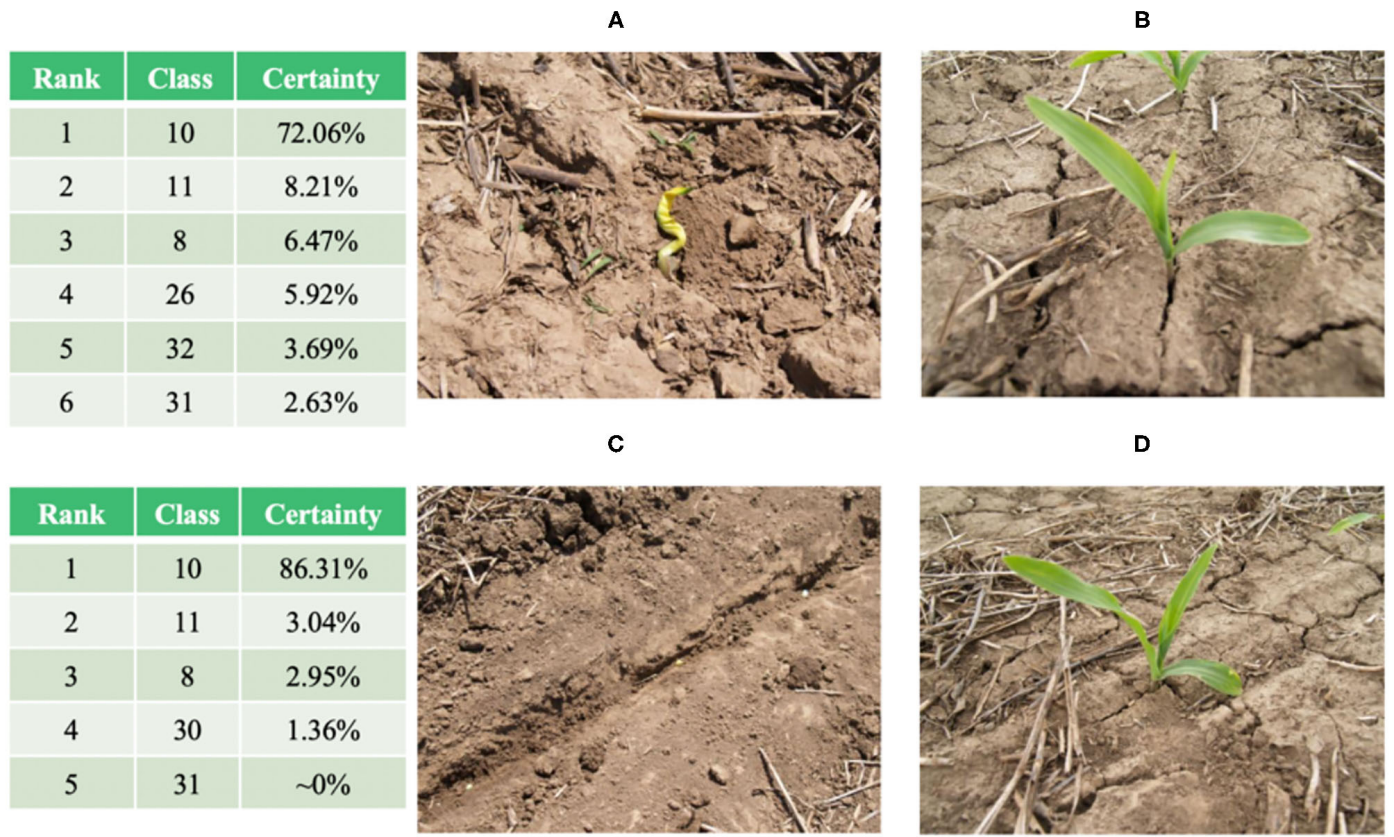
**(A,B)**: Images in category 31 and their corresponding “classification” results. **(C,D)**: representative images in category 10.

The fine-grained NASNetLarge model based on the attention mechanism reached an accuracy level of 95.62% on the test set of 11,640 images, of which 11,130 images were correctly classified. Among the 4.38% of misclassified images, there are some “problematic” images that do not contain crop leaves at all (as shown in Figures 8A,B). These images should be classified into category 31 (Tomato late blight, phytophthora infestans), but the model classifies their predictions into category 10 (Corn healthy), as shown in the classification table in Figure 8. The classification table shows the predicted classification ranking output of the final model on the original image. These images are misclassified by the model (the “correct” classification would be category 31). In fact, they do not belong to any category, because there are no crop leaves in the image. However, they are all classified as category 10. We infer that the images in category 10 (Figures 8C,D) contain similar soils, while the corn leaves are very slender and occupy a small portion of the image. If such problematic examples are excluded, the accuracy of the final model will be higher than 95.62%.

There are several other problems with the images obtained under field conditions, including: (1) shadows on the leaves in the images, with some images appearing dark and shaky; (2) other objects in the image that are not related to the leaf itself, such as a trunk, fruit, or fence. Note that these problematic images occupy a very small portion of the dataset. In short, according to the certainty levels provided by the final model, the attention mechanism-based approach proposed in this paper overcomes these problems in most cases.

**Figure 9:**
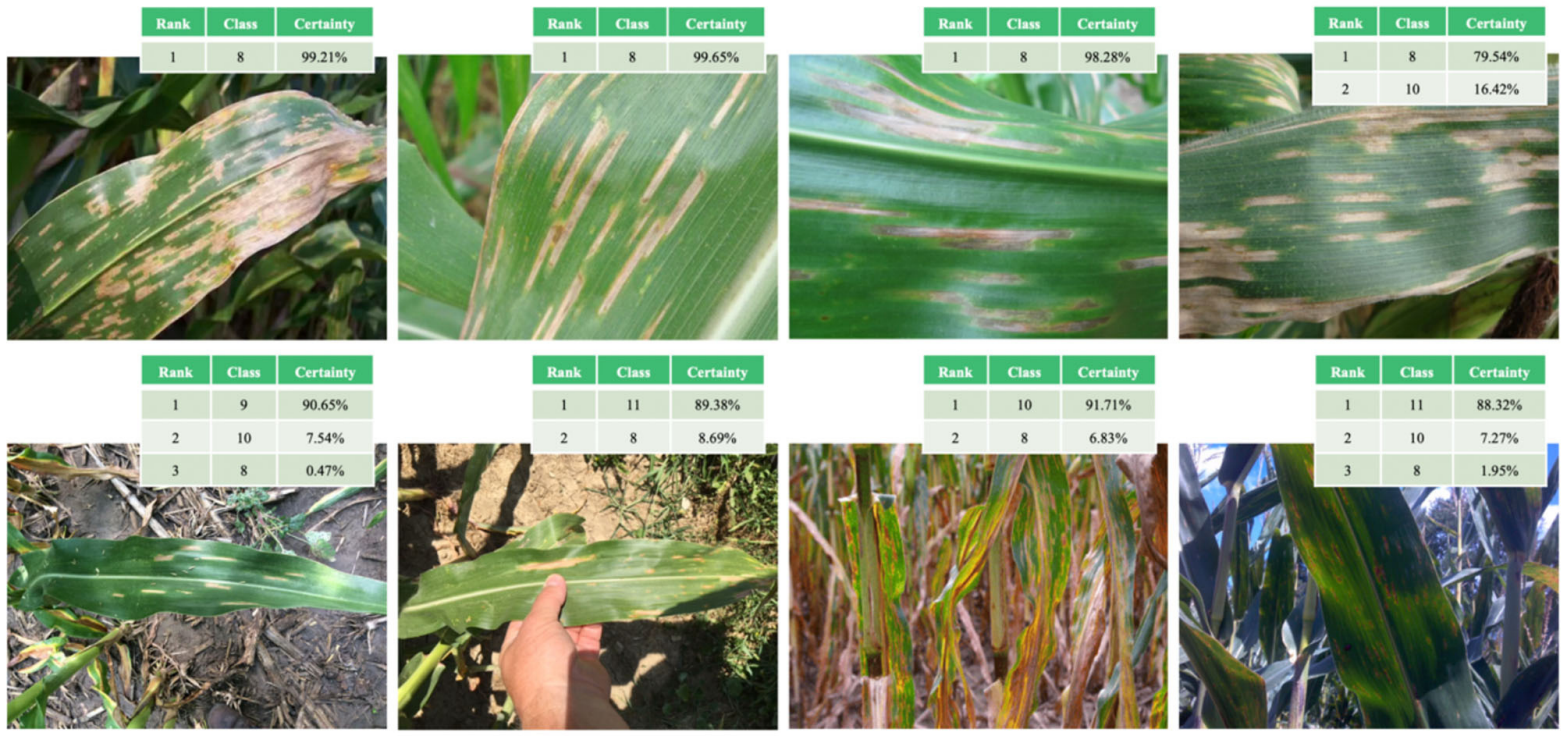
Examples of correct (upper four images) and incorrect (lower four images) classification of category eight images in the test set (*corn cercospora leaf spot*).

A typical case is category 8 (Corn cercospora leaf spot, cercospora zeae-maydis). Figure 9 shows the classification results of the model for eight representative images of category 8, including four incorrectly classified images (the lower four images in Figure 9) and four correctly classified images (the upper four images in Figure 9). The first three upper images were correctly classified, with a certainty of ~100%, while the fourth image was correctly classified with a certainty of ~79% (the second ranking, with a certainty level of 16% for category 10, corresponds to corn crops with different diseases). The four misclassified images (the lower four images in Figure 9) include a wide range of partial shadows or complex backgrounds, which increase the misclassification rate of the model. For the middle two lower images, the correct classification is ranked second, while the first ranking is category 10 or 11 (corn crop diseases). Therefore, the model correctly identifies the crop species, but does not accurately detect the particular crop disease.

## Conclusions

This study constructed, trained, and tested a fine-grained neural network model based on the attention mechanism for the classification of simple leaves of healthy or diseased crops. The model was trained using 58,200 publicly available images obtained under both laboratory conditions and field conditions. The data include 14 crop species in 37 different categories of [crop, disease] combinations, including some healthy crops. The optimal model was found to be a fine-grained NASNetLarge neural network based on the attention mechanism, which achieved an accuracy level of 95.62% (precision 94.35%, recall 91.79%, *F*_1_ value 93.05%) in the classification of the 11,640 images in the test set. The fine-grained NASNetLarge neural network model based on the attention mechanism achieves excellent classification performance by analyzing simple leaf images, so it is highly suitable for the automatic detection and diagnosis of crop diseases. In addition, the experimental results show that the images taken under field conditions in the training set are of high importance, indicating that when training such models, the proportion of images obtained under field conditions in the training set should be carefully considered.

For the backbone network of NASNetLarge, the results show that the NASNetMobile neural network model, similar to NASNetLarge, achieves state-of-the-art classification results on related datasets, surpassing the performance of previous lightweight networks such as MobileNet (Sandler et al., 2018) and ShuffleNet (Ma et al., 2018). As NASNetMobile requires little computing power to classify the given images, it can run on mobile devices such as smartphones, drones, or automatic agricultural vehicles for real-time monitoring and disease identification of large open-air crops. At present, due to the large-scale application of 5G, high-efficiency transmission, and improvements to the hardware configuration of mobile terminal equipment, it is possible to upload images locally to the cloud server for processing, and then return the identification and classification results to the terminal (Johannes et al., 2017; Toseef and Khan, 2018; Picon et al., 2019), or to use a GPU/CPU at the terminal to process and display the results (Barman et al., 2020). For growers in remote areas, real-time detection and diagnosis can be carried out through mobile terminals, thus solving the practical problems of obtaining technical crop disease diagnosis and finding experts in the production process. For agricultural technicians, this is equivalent to having a valuable auxiliary consultation tool. In the future, an intelligent crop disease prevention and control recommendation system will be developed based on the results of real-time diagnosis, allowing growers to select different prevention and control methods (e.g., physical or chemical methods) according to the specific conditions. The process and dosage of the methods will also be described in detail. Intelligent crop disease identification and diagnosis, as well as intelligent crop disease prevention and recommendation, will greatly improve production efficiency, realize agricultural, scientific, and technological progress, and push agriculture into the intelligent era.

Although the system developed in this study achieved a high success rate, it is far from becoming a universal tool under actual field conditions (Boulent et al., 2019). At present, the existing research has only considered dozens of [crop, disease] combinations (Ferentinos, 2018), so it is vital to expand the existing database to include more crop species and corresponding diseases. The test set used to evaluate the model was part of the dataset from which the training set was extracted, which is a potential source of bias. This is a common method for training and testing machine learning models. However, to develop a system that can be used effectively in field scenes, data from various sources should be used for testing to ensure that future users can obtain effective classification results in different scenes (Barbedo, 2018). At present, some preliminary experiments carried out with limited data show that when testing images different from those used in training, the classification performance of the model is significantly reduced to the range of 25–35%. The experimental results show that the classification effect depends on the data source. To improve this, more extensive image datasets should be collected from different geographical areas, field conditions, image capture modes, and multiple sources. Improving the model by increasing the size of the dataset would allow more effective and widespread identification of crop categories and diseases under field conditions.

## Data Availability Statement

The raw data supporting the conclusions of this article will be made available by the authors, without undue reservation.

## Author Contributions

GY and YH: conceptualization. GY: methodology, software, formal analysis, data curation, writing—original draft preparation, and visualization. GY and BX: validation. GY and YY: investigation. YH and YY: resources, supervision, and project administration. BX: writing—review and editing. All authors contributed to the article and approved the submitted version.

## Conflict of Interest

The authors declare that the research was conducted in the absence of any commercial or financial relationships that could be construed as a potential conflict of interest.
